# Supplementary material for: ERC 2.0 - evolutionary rate covariation update improves inference of functional interactions across large phylogenies
Source: bioRxiv. 2025 Feb 28:2025.02.24.639970. Preprint. [Version 1] doi: 10.1101/2025.02.24.639970 (PMC11888306; doi:10.1101/2025.02.24.639970)
Supplement: Supplement 1 [file NIHPP2025.02.24.639970v1-supplement-1.pdf]

bioRxiv preprint doi: https://doi.org/10.1101/2025.02.24.639970; this version posted February 28, 2025. The copyright holder for this preprint (which was not certified by peer review) is the author/funder, who has granted bioRxiv a license to display the preprint in perpetuity. It is made available under aCC-BY 4.0 International license.

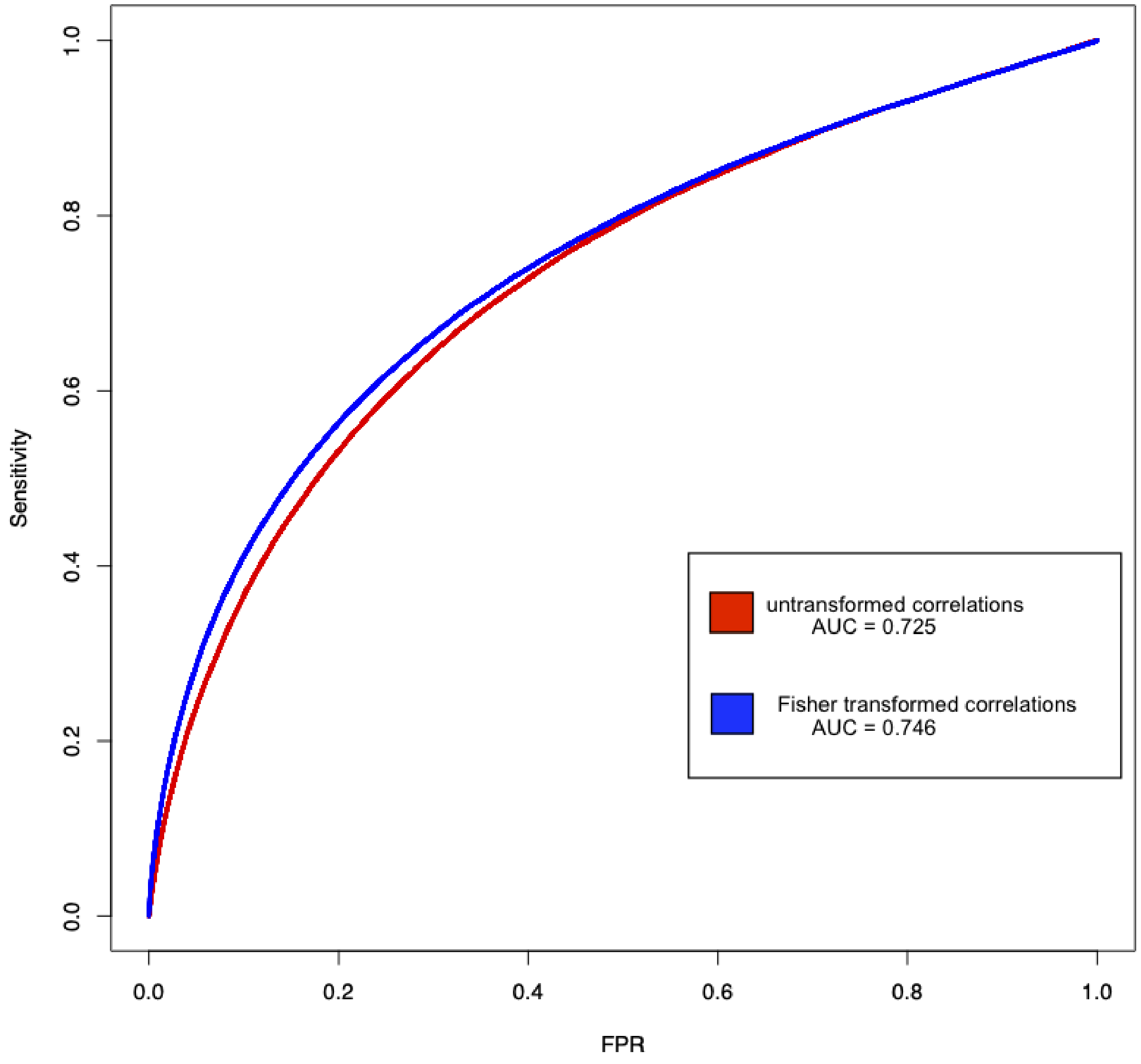

Figure S1

Fisher transformed correlations outperform untransformed Pearson correlations when comparing ERC to the STRING database. ROC curves comparing the predictive power of untransformed ERC correlations to Fisher transformed correlations for ERC calculated on 343 yeast when using the STRING database as the truth set. The red curve shows a ROC curve calculated on untransformed Pearson correlations and resulted in a ROC-AUC of 0.725. The blue line shows the ROC curve calculated using Fisher transformed Pearson correlations and resulted in a ROC-AUC of 0.746

bioRxiv preprint doi: https://doi.org/10.1101/2025.02.24.639970; this version posted February 28, 2025. The copyright holder for this preprint (which was not certified by peer review) is the author/funder, who has granted bioRxiv a license to display the preprint in perpetuity. It is made available under aCC-BY 4.0 International license.

Table S1:

Number of species (500 genes)	ERC2.0 run time (hr: min)	ERC1.0 run time (hr: min)
49	00:01	00:05
98	00:01	00:15
147	00:03	00:30
196	00:03	00:49
245	00:04	01:12
294	00:04	01:21
343	00:04	02:12

Run time using 10 CPUs on an x86_64 node

bioRxiv preprint doi: https://doi.org/10.1101/2025.02.24.639970; this version posted February 28, 2025. The copyright holder for this preprint (which was not certified by peer review) is the author/funder, who has granted bioRxiv a license to display the preprint in perpetuity. It is made available under aCC-BY 4.0 International license.

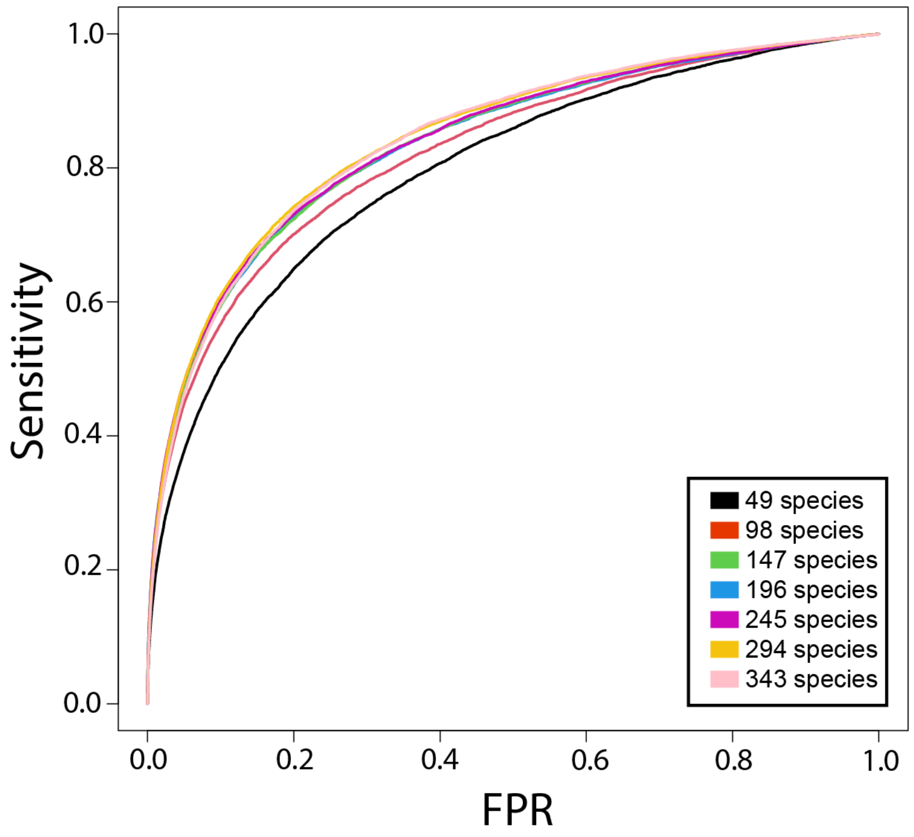

Figure S2:

ERC2.0 power to predict STRING interactions increases with species number until a plateau at 147 species.

ROC curves for increasingly larger subsets of yeast species from the whole dataset of 343 yeast species. Each subset increases by 49 species from the previous. ROC curves were calculated using STRING interactions as the truth set. FPR = false positive rate

bioRxiv preprint doi: https://doi.org/10.1101/2025.02.24.639970; this version posted February 28, 2025. The copyright holder for this preprint (which was not certified by peer review) is the author/funder, who has granted bioRxiv a license to display the preprint in perpetuity. It is made available under aCC-BY 4.0 International license.

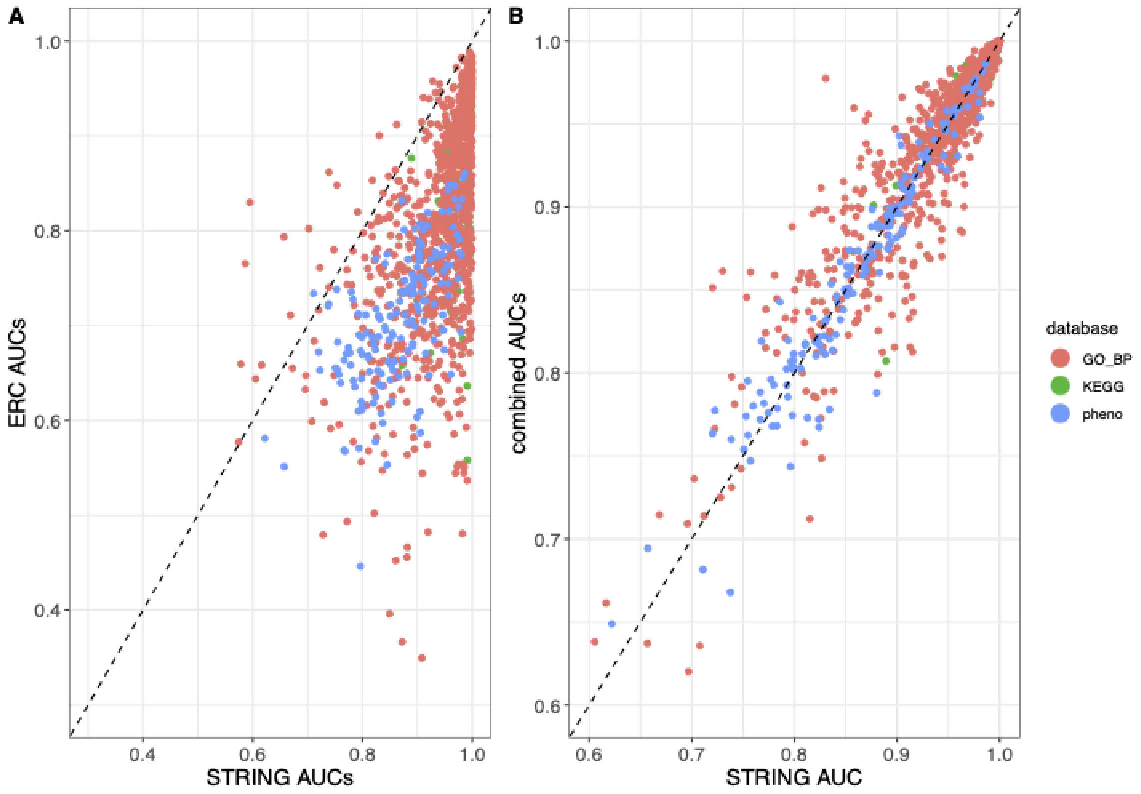

Figure S3:

Logistic regression models generated with STRING scores outperform those generated from ERC2.0 scores in yeast.

A. Scatterplot comparing the cv.glmnet lambda.1se AUC values using either STRING (x-axis) or ERC (y-axis) as the input data for three datasets in yeast: KEGG (green), Phenotypes (blue), and GO: Biological processes (red). Thirty annotation terms are above the diagonal, 29 from GO: Biological processes and 1 from phenotypes.

B. Scatterplot comparing the cv.glmnet lambda.1se AUC values using either STRING (x-axis) or combined STRING/ERC (y-axis) as the input data for three datasets in yeast: KEGG (green), Phenotypes (blue), and GO: Biological processes (red).

bioRxiv preprint doi: https://doi.org/10.1101/2025.02.24.639970; this version posted February 28, 2025. The copyright holder for this preprint (which was not certified by peer review) is the author/funder, who has granted bioRxiv a license to display the preprint in perpetuity. It is made available under aCC-BY 4.0 International license.

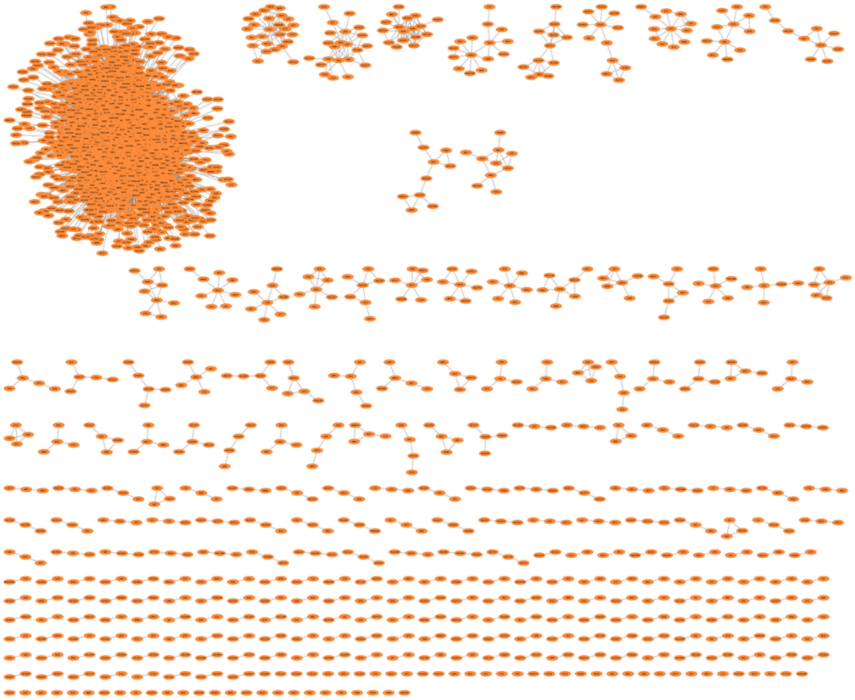

Figure S4:

ERC2.0 edges calculated using 343 yeast species are largely group in one cluster. MCL clustering of the top 500 thousand FtERC edges of the 343 yeast dataset. Each orange node represents a gene. The lines between nodes are ERC2.0 scores. Edges between clusters were removed for easier visualization.

bioRxiv preprint doi: https://doi.org/10.1101/2025.02.24.639970; this version posted February 28, 2025. The copyright holder for this preprint (which was not certified by peer review) is the author/funder, who has granted bioRxiv a license to display the preprint in perpetuity. It is made available under aCC-BY 4.0 International license.

Table S2:

Annotation term	Predicted member(s)
Oxidative phosphorylation	MME1
Ribosome	OG5228
Maturation of SSU-rRNA from tricistronic rRNA transcript	RPA190
Ribosomal large subunit biogenesis	UTP14, BMS1, MRD1, RPA190
translation	RPO41, MAM33, MBA1, YGR021W, OG4591
DNA-dependent DNA replication	MEC1
RNA splicing, via transesterification reactions with bulged adenosine as nucleophile	PRP8, PRP5, PRP28, OG4742
Cell wall mannoprotein/glycoprotein biosynthetic process	YJL181W
mRNA splicing, via spliceosome	PRP8, PRP5, MUD2, PRP28, OG4742, OG5197
Mitochondrial respiratory chain complex IV assembly/biogenesis	YLR283W
DNA-templated transcription, elongation	TAF1
mRNA processing	PRP5, PRP28
Respiratory electron transport chain	YMR166C
Pre-replicative complex assembly involved in nuclear cell cycle DNA replication	OG4420
phosphorylation	OG3228, OG4757
Regulation of transcription from RNA polymerase II promoter	TAF5, BDF1, UBP2, OG7669
Mitotic recombination	RFA1, RFA2
Septum formation	OG7034

bioRxiv preprint doi: https://doi.org/10.1101/2025.02.24.639970; this version posted February 28, 2025. The copyright holder for this preprint (which was not certified by peer review) is the author/funder, who has granted bioRxiv a license to display the preprint in perpetuity. It is made available under aCC-BY 4.0 International license.

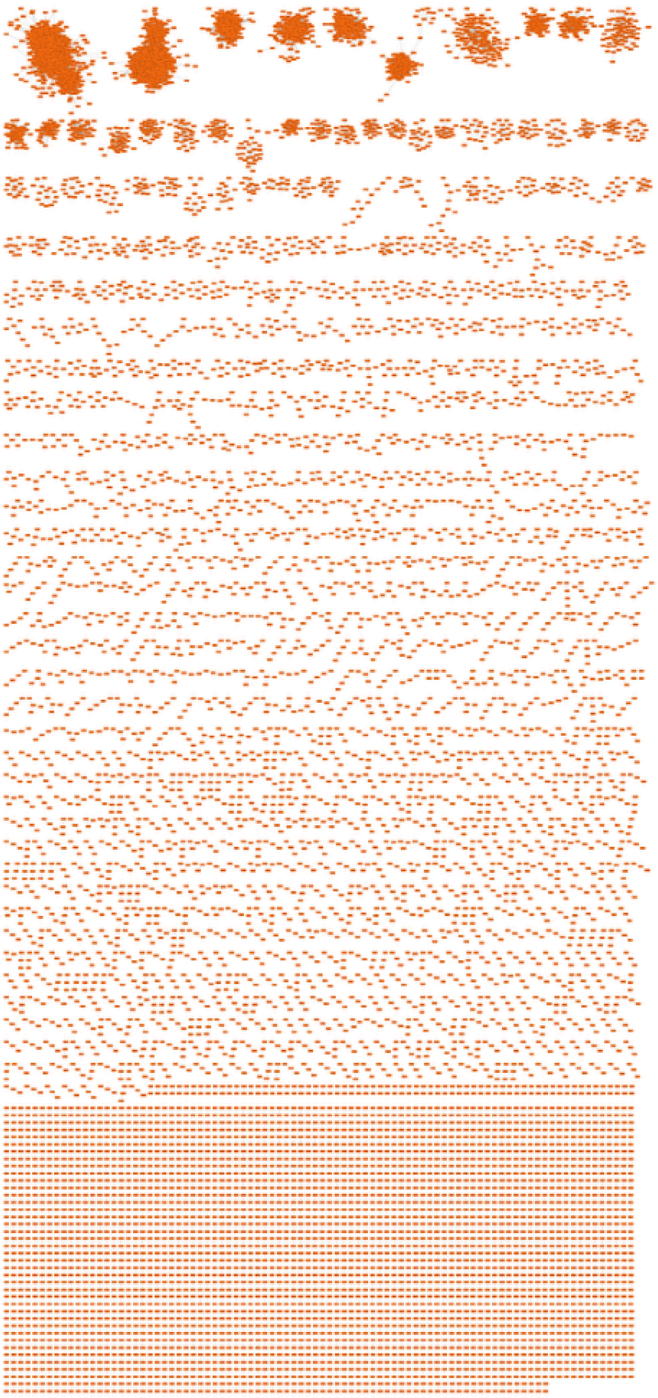

Figure S5:

MCL clustering of the top 500K FtERC edges in the mammal dataset
ERC2.0 edges calculated using 120 yeast species have many subclusters. MCL clustering of the top 500 thousand FtERC edges of the 120 mammal dataset. Each orange node represents a gene. The lines between nodes are ERC2.0 scores. Edges between clusters were removed for easier visualization.

bioRxiv preprint doi: https://doi.org/10.1101/2025.02.24.639970; this version posted February 28, 2025. The copyright holder for this preprint (which was not certified by peer review) is the author/funder, who has granted bioRxiv a license to display the preprint in perpetuity. It is made available under aCC-BY 4.0 International license.

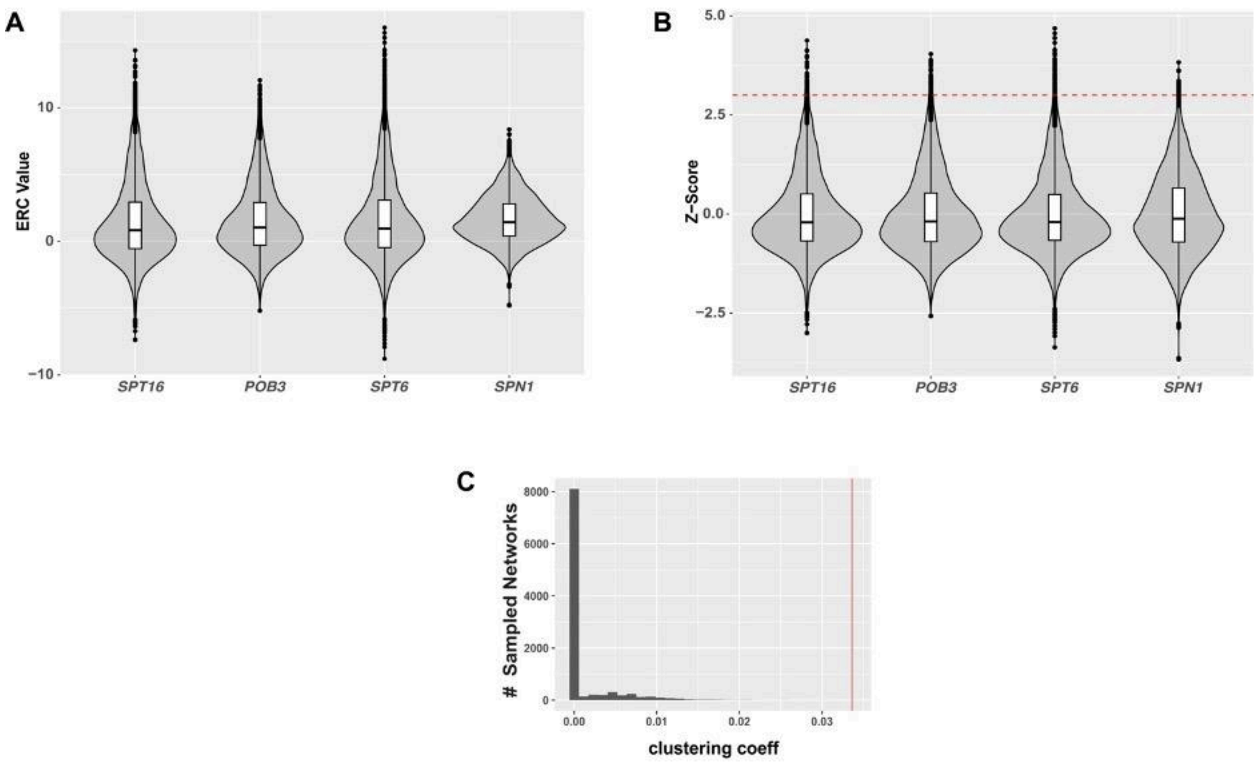

Figure S6:

The transcription-associated histone chaperone ERC network has a higher global clustering coefficient than randomly sampled networks. A) Violin plot showing the distribution of ERC values for *SPT16*, *POB3*, *SPT6*, and *SPN1*. B) Violin plot showing the distribution of Z-score standardized ERC values for *SPT16*, *POB3*, *SPT6*, and *SPN1*. The red line indicates a Z-score of 3, which was used as the cut-off examine at gene pairs with high ERC values. C) Histogram of the distribution of global clustering coefficients within 10,000 randomly sampled networks. Each sampled network was generated first by randomly choosing 4 query genes. For each set of query genes, a network was prepared as in Figure 7 using the genes with the top N ERC values for the respective query where N = 72, 67, 86, or 25, thus producing networks with the same total number of edges per query node. Displayed is the global connectivity of each sampled graph as assessed using the global clustering coefficient. Vertical red line indicates global clustering coefficient of the chaperone network in Figure 7 (0.033).
